# Mutational profile of Brazilian lung adenocarcinoma unveils association of *EGFR* mutations with high Asian ancestry and independent prognostic role of *KRAS* mutations

**DOI:** 10.1038/s41598-019-39965-x

**Published:** 2019-03-01

**Authors:** Letícia Ferro Leal, Flávia Escremim de Paula, Pedro De Marchi, Luciano de Souza Viana, Gustavo Dix Junqueira Pinto, Carolina Dias Carlos, Gustavo Noriz Berardinelli, José Elias Miziara, Carlos Maciel da Silva, Eduardo Caetano Albino Silva, Rui Pereira, Marco Antonio de Oliveira, Cristovam Scapulatempo-Neto, Rui Manuel Reis

**Affiliations:** 1 Molecular Oncology Research Center, Barretos Cancer Hospital, Barretos, Brazil; 2 Center of Molecular Diagnoses, Barretos Cancer Hospital, Barretos, Brazil; 3 Medical Oncology Department, Barretos Cancer Hospital, Barretos, Brazil; 4 Department of Thoracic surgery, Barretos, Brazil; 5 Department of Pathology, Barretos Cancer Hospital, Barretos, São Paulo Brazil; 6 Institute of Research and Innovation in Health, University of Porto, Porto, Portugal; 7 Institute of Molecular Pathology and Immunology at the University of Porto (IPATIMUP), Porto, Portugal; 8 Statistics Unity, Barretos Cancer Hospital, Barretos, São Paulo Brazil; 9 Life and Health Sciences Research Institute (ICVS), Health Sciences School, University of Minho, Braga, Portugal; 10 ICVS/3B’s-PT Government Associate Laboratory, Braga/Guimarães, Portugal

## Abstract

Lung cancer is the deadliest cancer worldwide. The mutational frequency of *EGFR* and *KRAS* genes in lung adenocarcinoma varies worldwide per ethnicity and smoking. The impact of *EGFR* and *KRAS* mutations in Brazilian lung cancer remains poorly explored. Thus, we investigated the frequency of *EGFR* and *KRAS* mutations in a large Brazilian series of lung adenocarcinoma together with patients’ genetic ancestry, clinicopathological and sociodemographic characteristics. The mutational frequency of *EGFR* was 22.7% and *KRAS* was 20.4%. The average ancestry proportions were 73.1% for EUR, 13.1% for AFR, 6.5% for AME and 7.3% for ASN. *EGFR* mutations were independently associated with never-smokers, high-Asian ancestry, and better performance status. *KRAS* mutations were independently associated with tobacco exposure and non-Asian ancestry. *EGFR*-exon 20 mutations were associated with worse outcome. The Cox regression model indicated a worse outcome for patients whose were older at diagnosis (>61 y), solid histological subtype, loss of weight (>10%), worse performance status (≥2), and presence of *KRAS* mutations and *EGFR* mutational status in TKi non-treated patients. In conclusion, we assessed the clinicopathological and ethnic impact of *EGFR* and *KRAS* mutations in the largest series reported of Brazilian lung adenocarcinomas. These findings can support future clinical strategies for Brazilian lung cancer patients.

## Introduction

Lung cancer is the deadliest cancer worldwide and in Brazil^1–3^. The 5-year survival rate for lung cancer patients is lower than 20%, possibly due to the lack of successful early detection and limited treatment options^4^. Over the past decade, the treatment of advanced/metastatic non–small cell lung cancer (NSCLC) has experienced significant modification mainly due to development of molecular testing to determine a druggable mutation and due to the addition of immune-based therapies^5,6^. Histologically, lung cancer is divided into non-small cell lung cancer (NSCLC), which corresponds about 85% of all lung cancer cases and, less commonly, small cell lung cancer (SCLC). The most common histologic subtype of NSCLC is adenocarcinoma^7^.

Epidermal growth factor receptor (*EGFR*) gene has a key role in the pathogenesis of several tumors^8–10^. Activating *EGFR* mutations are involved in the pathogenesis of a significant subset of lung adenocarcinomas^11^. The frequency of *EGFR* mutations in lung adenocarcinomas worldwide varies (from 8–13% in European populations to 27–60% Asian populations) according to ethnicity, gender, and tobacco exposure^12,13^. *EGFR* gene has emerged as a critical therapeutic target and *EGFR* mutations status has successfully guided clinical management^14^. The presence of activating *EGFR* mutations, mainly in exons 18, 19, 20 and 21, which correspond to the tyrosine kinase domain, sensitizes lung adenocarcinomas to treatment with anti-EGFR tyrosine kinase inhibitors (TKi), such as erlotinib, gefitinib, afatinib, dacomitinib and osimertinib^15,16^. However, basically, all TKi-treated lung adenocarcinoma patients will experience disease progression due to resistance mechanisms^17,18^. The most well-known resistance mechanism is the presence of *EGFR* p. T790M mutation and patients harboring this mutation are eligible for treatment with second and third generation of anti-EGFR TKi, such as osimeritinib^19^.

Another important oncogene in lung cancer is the *KRAS* (Kirsten rat sarcoma viral oncogene homolog), which codifies an EGFR downstream GTPase^20^. Hotspot *KRAS* mutations, at codons 12/13, are described in approximately 20% lung adenocarcinomas and are known to be associated with tobacco consumption^21^. Since *KRAS* and *EGFR* mutations are mutually exclusive events, molecular testing based on *KRAS* mutational status for treatment decisions was not currently recommended for anti-*EGFR* TKi^22^. However, combined strategies for targeting *KRAS* mutations can be promising^23^. Moreover, *KRAS* mutational status was described to be associated with the immune microenvironment implying that the molecular testing for this gene should be considered for stratifying patients aimed at immunotherapy^24^.

Taken together, the molecular testing of *EGFR* and *KRAS* mutations for lung cancer patients has offering advantages for guiding personalized therapy, providing a better patient selection and stratification for clinical management.

Reports of the prevalence of *EGFR* and *KRAS* mutations among Brazilian patients remain limited^25,26^. Moreover, it is also poorly explored the impact of *EGFR* and *KRAS* mutations in patients’ clinicopathological features. Therefore, the present study aims to investigate the frequency of *EGFR* and *KRAS* mutations in a large Brazilian series of lung adenocarcinoma, and to correlate the presence of these mutations with patients’ genetic ancestry and clinicopathological and sociodemographic characteristics.

## Results

### *EGFR* and *KRAS* mutational status

**Figure 1:**
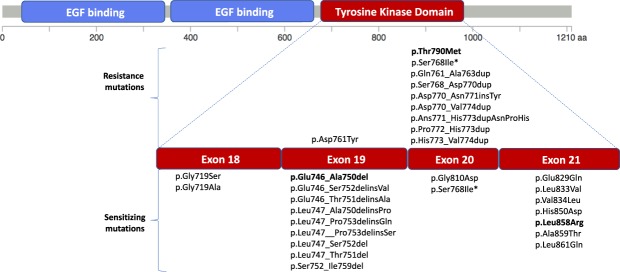
*EGFR* mutations and exon distribution. EGFR protein structure highlighting the tyrosine kinase domain (red), which is the hotspot region (red) for the *EGFR* mutations with predictive value. All resistance and sensitizing mutations detected in the present series of Brazilian lung adenocarcinoma are presented. *Controversial results in the literature.

*EGFR* mutations were detected in 101 patients (22.7%) and mutations were distributed at tyrosine kinase domain predominantly located at exons 19 and 21 (Fig. 1 and Supplementary Fig. 1 and Supplementary Table 1). The most common *EGFR* mutation was the p. Leu858Arg (n = 32) followed by a substitution the p. Glu746_Ala750del (n = 31; Supplementary Fig. 1 and Supplementary Table 1). Additional *EGFR* sensibility mutations include mutations located at exons 18 (p. Gly719Ser and p. Gly719Ala), 19 (p.Glu746_Ser752delinsVal, p.Glu746_Thr751delinsAla, p.Leu747_Ala750delinsPro, p.Leu747_Pro753delinsGln, p.Leu747__Pro753delinsSer, p.Leu747_Ser752del, p.Leu747_Thr751del, p.Ser752_Ile759del), 20 (p. Gly810Asp) and 21 (p.Glu829Gln, p.Leu833Val, p.Val834Leu, p.His850Asp, p.Leu858Arg, p.Ala859Thr, p.Leu861Gln) (Fig. 1 and Supplementary Table 1).

Among *EGFR* positive patients, 12 presented mutations associated with drug resistance, namely p.Tyr790Met the most common one (n = 3; exon 20), followed by p.Gln761_Ala763dup (n = 2; exon 20), p.Asp761Tyr (n = 1; exon 19), p.Ser768_Asp770dup, p.Asp770_Asn771insTyr, p.Asp770_Val774dup, p.Ans771_His773dupAsnProHis, p.Pro772_His773dup and p.His773_Val774dup (n = 1/each; exon 20) (Supplementary Table 1 and Fig. 1). The p.Ser768Ile mutation has been described as both sensitivity and resistance mutation depends on the used drug (Fig. 1)^27,28^. Among the p. Tyr790Met-mutated cases, 2 of them were concomitant with p.Leu858Arg and p.Leu861Gln mutations.

Furthermore, we also tested a real time-PCR based commercial assay Cobas^®^ *EGFR* Mutation Test (Roche), in the cases that exhibited uncommon mutations, namely, the p.Leu861Gln, p.Ans771_His773dupAsnProHis, p.Asp770_Val774dup, p.Pro772_His773dup, and p.His850Asp. The COBAS assay showed an absence of a mutation in those samples. Of note, the p.Leu861Gln was later included in the expanded version Cobas^®^ *EGFR* Mutation v2 Test.

*KRAS* mutations (codons 12/13) were detected in 90 patients (20.4%) (Supplementary Fig. 1 and Supplementary Table 2). The most common *KRAS* mutation was p.Gly12Cys (n = 32), followed by p.Gly12Val (n = 21), p.Gly12Asp (n = 19), p.Gly13Cys (n = 6), p.Gly12Ala (n = 5), p.Gly12Ser (n = 2) and p.Gly12Phe, p.Gly13Asp, p.Gly13Glu, p.Gly13Ser and p.Gly13Val (n = 1/each) (Supplementary Table 2**)**.

All *EGFR* and *KRAS* mutations were mutually exclusive.

### Genetic ancestry component and its association with *EGFR* and *KRAS* mutations

**Figure 2:**
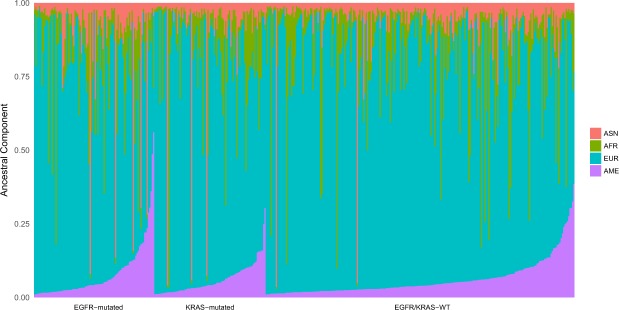
Individual ancestry proportion of Brazilian lung adenocarcinoma series (n = 427 out of 444). The pinkish-orange, green, blue and purple colors represent the Asian, African, European and Native American ancestry proportions, respectively.

We further assessed the ancestry background by an AIM-INDEL panel that allowed to estimates the AFR, EUR, ASN and AME ancestral proportions in 427 out of 444 patients (Fig. 2). The average ancestry proportions for all individuals were 73.1% for EUR, 13.1% for AFR, 6.5% for AME and 7.3% for ASN (Fig. 2). Ancestry proportions were further categorically defined as low, intermediate and high based on terciles (Supplementary Table 3). Most of our patients were self-declared as white (Table 1) and likewise most of our cases presented high EUR background (Fig. 2).

**Table 1:** Major clinicopathological features of NSCLC adenocarcinomas.

Variables	Parameters	n
Age^a^, years	61 (22–87)	444
Gender	Male	232
	Female	212
Self-reported race^b^	White	342
	Brown	64
	Black	20
	Yellow	7
Smoking history	Never smoker	135
	Current Smoker	171
	Former smoker	131
Alcohol consuming	Never	272
	Current	114
	Former	35
Metastasis at diagnosis	No	114
	One site	129
	Multiple sites	200
Disease staging	I	28
	II	15
	III	71
	IV	329
PS ECOG	0	46
	1	233
	2	73
	3 or 4	88
Loss of weight^c^	No	209
	<10%	145
	>10%	69
Histology^d^	Adenocarcinoma NOS	120
	Acinar	163
	Solid	112
	Papillary	45
	Lepidic	12
	Mucinous	7

n, number of patients; PS ECOG, performance status ECOG (Eastern Cooperative Oncology Group).^*a*^Average age (range); ^*b*^Self-reported race according to Brazilian Institute of Geography and Statistics (IBGE). ^*c*^Loss of weight <10% and >10% of total body weight. ^*d*^Adenocarcinoma predominant subtypes; When histology subtype was not determined, the case was considered as only adenocarcinoma.

We then correlated the genetic ancestry with the molecular features. *EGFR* mutations were associated with high ASN (p = 0.03; Supplementary Table 4). In the multivariate analysis, the high ASN background was independently associated with the presence of *EGFR* mutations [OD = 2.01 (1.09–3.71); p = 0.03; Table 2]. On the other hand, in the multivariate analysis, the low ASN background was independently associated with the presence of *KRAS* mutations [OD = 1.93 (1.06–3.52); p = 0.03; Table 2].

**Table 2:** Multivariate analysis of the association between clinicopathological characteristics and ancestry background and *EGFR* and *KRAS* mutations.

	Variables	Parameters	Total (n)	OR	95% CI	p-value
*EGFR*	Gender	Male	224	1	Ref.	Ref.
		Female	196	1.67	0.98–2.85	0.058
	Tobacco	Never	126	5.11	2.71–9.62	**<0.0001**
		Current smoker	166	0.59	0.28–1.24	0.16
		Former smoker	128	1	Ref.	Ref.
	ASN Ancestry	Low	140	1	Ref.	Ref.
		Intermediate	138	1.05	0.54–2.04	0.88
		High	142	2.01	1.09–3.71	**0.03**
	PS ECOG	0	44	3.94	1.47–10.57	**0.006**
		1	222	1.67	0.79–3.53	0.18
		2	67	1.2	0.46–3.13	0.72
		3 or 4	84	1	Ref.	Ref
*KRAS*	Tobacco	Never	126	1	Ref.	Ref.
		Current smoker	166	3.42	1.67–7.00	**0.001**
		Former smoker	128	3.74	1.79–7.81	**<0.0001**
	ASN Ancestry	Low	140	1.93	1.06–3.52	**0.03**
		Intermediate	138	1.31	0.69–2.46	0.41
		High	142	1	Ref.	Ref.

n, number of patients; OR, odds ratio; 95% CI, 95% confidence interval; p-value: significance of Wald test.; ASN, Asian race; Ref., reference group; PS ECOG, performance status ECOG (Eastern Cooperative Oncology Group). Significant associations are indicated in bold.

The additional ethnic groups, AFR, EUR, and AME, were not associated with the presence of *EGFR* and *KRAS* mutations (Supplementary Table 4).

### Association of patients’ clinicopathological features and molecular features

**Figure 3:**
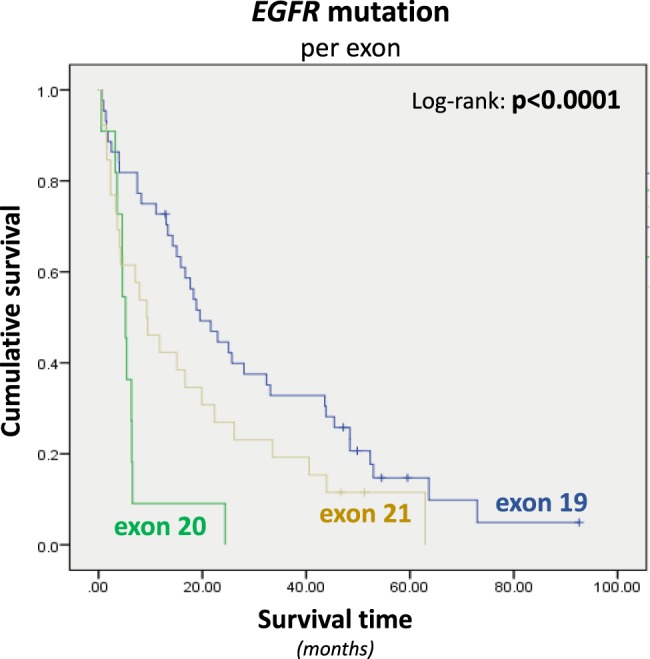
Kaplan-Meier curves for overall survival of lung adenocarcinoma patients (stage IV) according to *EGFR* mutations categorized by exon distribution (Median OS exon 19 = 19.5 months; Median OS exon 20 = 5.2 months; Median OS exon 21 = 9.3 months). Survival time is presented in months; p values are related to Log-rank test results. Exon 18 was not included in the analysis because only one patient with disease stage IV carries *EGFR* exon 18 mutation.

The presence of *EGFR* mutations was associated with female gender (p < 0.0001), absence of smoking habit (p < 0.0001), absence of alcohol consuming (p = 0.002); and better ECOG PS (p = 0.03) (Supplementary Table 4). Age, self-reported race, disease staging, metastasis at diagnosis, weight loss at diagnosis, and differentiation grade were not associated with the presence of *EGFR* mutations (Supplementary Table 4). *EGFR*-mutated patients harboring exon 20 mutations presented the lowest OS (exon 19 = 3.4 months; exon 20 = 0.5 months; exon 21 = 3.0 months; Fig. 3). In addition, *EGFR*-mutated patients harboring exon 20 mutations mostly presented with disease progression (exon 19 = 22.2%; exon 20 = 55.6%; exon 21 = 22.2%), only one patient harboring exon 20 mutation presented partial response to TKi (exon 19 = 85.3%; exon 20 = 2.9%; exon 21 = 11.8%) and none of the patients harboring exon 20 mutations presented with stable disease (exon 19 = 55.6%; exon 20 = 0%; exon 21 = 44.4%; p < 0.0001; Supplementary Table 5). Exon 18 was not included in the analysis because only one patient with disease stage IV carries *EGFR* exon 18 mutation.

A multivariate analysis indicated the following independent variables were associated with the presence of *EGFR* mutations: absence of smoking habit (OR = 5.11; p < 0.0001; Table 2), high ASN (OR = 2.01; p = 0.03; Table 2) and better ECOG PS (OR = 3.94; p = 0.006; Table 2). Female gender was marginally associated with the presence of *EGFR* mutations (OR = 1.67; p = 0.058; Table 2).

Concerning *KRAS* status, the presence of mutations was associated with the presence of smoking habit (p < 0.0001). Age, self-reported race, disease staging, metastasis at diagnosis, weight loss at diagnosis, and differentiation grade were not associated with the presence of *KRAS* mutations (Supplementary Table 4). A multivariate analysis indicated the following independent variables as associated with the presence of *KRAS* mutations: tobacco exposure (current: OR = 3.42; p = 0.001/former: OR = 3.74; p < 0.0001; Table 2) and low Asian ancestry (OR = 1.93; p = 0.03; Table 2).

Since most patients were diagnosed at disease stage IV, and they exhibit a very distinct outcome from stage I, II and III (Supplementary Fig. 2), we only analyzed this group of patients in the multivariate analysis of disease outcome (Table 3). Unfavorable outcome was independently associated with age at diagnosis higher than 61 years old (OR = 1.45; p = 0.01), solid histological subtype (OR = 1.91; p < 0.0001), increased weight loss (OR = 1.72; p = 0.006), ECOG PS 2 and 3 or 4 (OR = 2.43 and OR = 6.28, respectively; p = 0.03 and p < 0.0001, respectively) (Table 3). Metastasis at diagnosis in the central nervous system was considered a risk factor for an unfavorable outcome (Table 3). Clinical outcome was not independently associated with self-reported race and alcohol consuming (Table 3).

**Table 3:** Multivariate analysis of the association between clinicopathological characteristics and overall survival.

Variables	Parameters	Total (n)	OR	95% CI	p-value
Age*	≤61 years	126	1	Ref.	Ref.
	>61 years	107	**1.45**	1.09–1.93	**0.01**
Histology	Acinar	119	1	Ref.	Ref.
	Mucinous	5	0.77	0.27–2.20	0.63
	Lepidic	8	1.01	0.43–2.39	0.98
	Papillary	27	1.22	0.76–1.97	0.41
	Solid	74	**1.91**	1.36–2.68	**<0.0001**
Loss of weight**	No	105	1	Ref.	Ref.
	<10%	81	1.11	0.80–1.53	0.55
	>10%	47	**1.72**	1.17–2.54	**0.006**
PS ECOG	0	13	1	Ref.	Ref
	1	122	1.64	0.78–3.46	0.16
	2	42	**2.43**	1.07–5.51	**0.03**
	3 or 4	56	**6.28**	2.80–14.08	**<0.0001**
Metastasis at diagnosis	CNS	74	1	Ref.	Ref.
	Other sites	159	**0.62**	0.45–0.85	**0.004**
TKi_*EGFR*	Yes_WT	13	1	Ref.	Ref.
	Yes_Mutated	48	0.91	0.45–1.84	0.926
	No_WT	152	1.75	0.92–3.31	0.052
	No_Mutated	20	**3.79**	1.73–8.33	**0.001**
*KRAS* mutations	WT	194	1	Ref.	Ref.
	Mutated	39	**2.93**	1.94–4.42	**<0.0001**

*Only patients diagnosed at stage IV were included in this analysis. n, number of patients; OR, odds ratio; 95% CI, 95% confidence interval; p-value: significance of Cox Regression; Ref., reference group; *age was categorized into two groups considering the average age of the entire series as the cutoff; **Loss of weight <10% and >10% of total body weight; PS ECOG, performance status ECOG (Eastern Cooperative Oncology Group); CNS, central nervous system; TKi_EGFR, combination of two variables (TKi treatment and EGFR mutation). Significant associations are indicated in bold.

The Cox regression analysis indicated the presence of *EGFR* mutations in TKi non-treated patients were independently associated with unfavorable outcome (OR = 3.79; p = 0.001; Median OS TKi non-treated = 6.7 months; Median OS TKi-treated = 19.9 months) (Table 3 and Supplementary Fig. 2). Of note, among the *EGFR-*mutated patients that were not treated with TKi (n = 30), 15 received Best Support of Care (BSC), 9 received only chemotherapy, and 6 of them had localized disease and they were submitted to local treatment.

In addition, the Cox regression analysis also showed that the presence of *KRAS* mutations was independently associated with unfavorable outcome (OR = 2.93; p < 0.0001; Table 3).

## Discussion

Genetic testing is crucial for molecular-targeted therapies in NSCLC. In Brazil, less than half of the Brazilian cancer patients are tested for therapeutic targets and the public health system does not cover the greatest majority of molecular testing for NSCLC^29^. Conversely, at the Barretos Cancer Hospital, a non-profit cancer center where 100% of patients are from the public health system^30^, all non-squamous NSCLC patients are benefited with the molecular testing for tailored therapies. Herein, we reported the association of *EGFR* and *KRAS* mutational status with clinicopathological features from approximately 500 Brazilian lung adenocarcinoma patients attended at the Barretos Cancer Hospital.

Overall, regarding clinical and histopathological characteristics, adenocarcinoma solid subtype was strongly associated with worse disease outcome in a multivariate analysis irrespective the presence of *EGFR* mutations. Since only a subset of tumors was suitable for histological subclassification, few histological subgroups have a small number of cases. Nevertheless, solid histological subtype had been previously associated with a worse prognosis^25,31^.

Although the role of *EGFR* has been well established in the last few years, data on Brazilian populations remains limited. In the present study, the frequency of *EGFR* mutations was 22.7%. Previous findings from smaller Brazilian cohorts described frequencies of *EGFR* mutations between 22% (27/125) to 30% (63/207)^25,26^. The most recurrently *EGFR* mutation in the present adenocarcinoma series was a deletion in exon 19 followed by a substitution in exon 21 (p.L858R), similarly to previously reported in Brazilian patients^25^. These mutations are known to be sensitive to TKi. Interestingly, in the present study, the *EGFR*-mutated patients that were not treated with TKi, due to poor PS at diagnosis or death before receive the result of molecular test, presented a worse outcome compared with those *EGFR*-mutated patients TKi-treated, supporting the clinical benefit of TKi in *EGFR* mutated patients. As expected, the most recurrently *EGFR* resistance mutation was the p. Tyr790Met and further *EGFR* resistance mutations were mostly located at exon 20^32^. In accordance, *EGFR*-mutated patients harboring mutations located at exon 20 presented lower overall survival and compromised response to TKi.

Several commercial assays are available for *EGFR* testing, such as COBAS (Roche), and Therascreen (Qiagen) among others, which are realtime-PCR based and are built to harbor the major mutations reported in the literature. In this context, we can hypothesize that approximately 13% of the mutations identified in our series, mainly located in exon 20 and 21, would not be detected by these commercial assays that are widely used. Thus, these results emphasize the importance of the knowledge of the mutational profile of each population to better guide the methodology used for routine practice.

We next interrogate the impact of *EGFR* status in patients’ clinicopathological features. In a multivariate analysis, we observed that *EGFR* mutated cases was associated with never smokers, better PS, and higher Asian ancestry, and a tangentially with the female gender. These results are in accordance with the literature^16,26,33–35^. Interestingly, the association of *EGFR* mutation with higher Asian ancestry observed in our Brazilian cases is related with the admixture of Asian background in our Brazilian cases, probably due to Japanese/Korean/Chinese immigration wave in the 1940’s.

We observed *KRAS* mutations in 20.4% of lung adenocarcinomas. This frequency is in accordance with reported in international literature, that vary from 15–33% of cases^21,33,34,36^. Likewise, our results are in line with the two previous Brazilian reports, which showed 15% (30/207) and 26% (33/115) of *KRAS* mutations in lung cancer^25,26^. As well reported^26^, we found that *KRAS* mutations were more frequently found in patients who reported tobacco exposure. We also observed an independent association of *KRAS* mutation with a lower Asian background. Importantly, in our series, *KRAS* mutation was an independent factor for unfavorable outcome supporting the prognostic value of *KRAS* mutations. The prognostic role of *KRAS* in lung cancer is not consensual, with diverge reports^37,38^. Recently, it was reported that *KRAS* mutation induced upregulation of PD-L1, through p-ERK, mediated immune escape in lung adenocarcinoma, and induces the apoptosis of CD3-positive T cells, which were reversed by anti-PD-L1 or ERK inhibition^39^. In addition, it was reported that specific *KRAS* mutations could affect the immune microenvironment of lung adenocarcinoma patients, which affect the efficacy of immune checkpoint inhibitors, implying stratification of patients for immunotherapy should be tailored based on the specific mutant *KRAS* variants and tumor microenvironment^24^. Thus, following the advent of immunotherapy, *KRAS* mutations have rewarded new purposes with the promising clinical utility.

The present study harbors some limitations, mainly due to the retrospective nature of the study, therefore patients were not treated uniformly, which hamper proper outcome analysis and only patients diagnosed at stage IV were included in the survival analysis, since patients diagnosed at stages I, II, and III presented distinctive outcomes compared with stage IV.

Concluding, this is the largest study assessing *EGFR* and *KRAS* mutation status in the Brazilian lung adenocarcinoma population. *EGFR* mutation was associated with Asian ancestry background, confirming the known geographic disparities. In our series, *KRAS* mutation was an independent prognostic factor. Overall, these data provide important information about the role of some of the most important driver genes and tailored-guided treatment for lung adenocarcinoma in the Brazilian population.

### Materials e Methods

#### Study population and design

This is a retrospective study conducted at the Center for Molecular Diagnosis, from patients diagnosed at Barretos Cancer Hospital from 2011 to 2014. Overall, 496 NSCLC cases, who underwent surgical resection or core biopsy were histopathologically re-evaluated. Of these, 52 cases with non-adenocarcinoma histology were excluded for further analysis. A subset of this series was previously published and tested for *ALK* translocations^40^. The major clinicopathological features of the 444 lung adenocarcinomas are summarized in Table 1. Overall, 232 were male (52%) and 212 female (48%) with an average age at the diagnosis of 61 years old (22–87 years). Most of the patients were self-reported as white (79%), were current or former smokers (77%) and were no alcohol consumers (61%). Most patients were diagnosed at stage IV (74%) and among these patients, most of them presented metastasis at multiple sites (61%). ECOG PS 1 was the most prevalent at diagnosis, and weight loss was observed in half of the patients (50.6%). The most predominant histological subtype was acinar, followed by solid, papillary, lepidic and mucinous but about a quarter of the tumors (27%) was not possible to determine the histological subtype (Table 1).

Considering the present study enrolls a retrospective series, patients were treated ununiformly. Detailed information about treatment regimens are described in supplementary material (Supplementary Tables 6–9).

The present study was approved by the Barretos Cancer Hospital IRB (Project n°. 630/2012), which bestowed the exemption of informed consent due to the retrospective nature of the study since most of the patients are dead. All methods were performed in accordance with the relevant guidelines and regulations.

#### DNA isolation

Serial 10 μm unstained sections of FFPE blocks were cut for DNA isolation and one hematoxylin and eosin-stained (H&E) section was taken for pathological evaluation and selection of the tumor area as previously reported^41^. Briefly, sections were heated at 80 °C and serial washes with xylene and ethanol (100, 70 and 50%) were performed for paraffin removal. Then, sections were macrodissected using a sterile needle and carefully collected into a microtube. Next, DNA was isolated from FFPE tissues using the QIAmp DNA micro kit (Qiagen, Hilden, Germany) following the manufacturer’s instructions. DNA concentration and quality were evaluated by Nanodrop 2000 (Thermo Scientific, Wilmington, USA). DNA samples were diluted to a final concentration of 50 ng/μL and stored at −20 °C for further molecular analysis.

#### Mutational analysis for *EGFR* and *KRAS* hotspot regions

The mutational analysis for hotspots regions of *EGFR* (exons 18, 19, 20 and 21) and *KRAS* (exon 2, codons 12 and 13) genes was analyzed by PCR, followed by direct sequencing, as previously described^9,42^. Briefly, the PCR reaction was performed with 50 ng of DNA in a final volume of 15 µL, using 10 µM of both forward and reverse primers and 7,5 µl of the HotStart master mix (Qiagen, Hilden, Germany), following the manufacturer’s instructions. PCR conditions are following described: 96 °C for 15 minutes, 40 cycles of 96 °C for 45 seconds, 56.5 °C for 45 seconds, 72 °C for 45 seconds and a final extension of 72 °C for 10 minutes.

The amplification of PCR products was checked by electrophoresis in agarose gel and purified by enzymatic reaction (ExoSAP-it, ThermoFisher Scientific). Next, direct sequencing was carried out using BigDye Terminator v3.1 Cycle Sequencing kit (ThermoFisher Scientific) with the following conditions: 97 °C for 3 minutes, 28 cycles of 96 °C for 10 seconds, 50 °C for 5 seconds, and 60 °C for 4 minutes. Sequencing products were purified using BigDye Xterminator (ThermoFisher Scientific) and analyzed on a 3500 Genetic Analyzer, ABI capillary electrophoresis system (Applied Biosystems). Sequences were captured by the SeqScape software (Applied Biosystems) and manually compared to reference sequences collected from GenBank (*EGFR*: NG_007726.3; *KRAS*: NG_007524.1). All mutations were confirmed twice.

#### Cobas^®^*EGFR* Mutation

A subset of cases was processed using the Cobas^®^ DNA Sample Preparation Kit for manual sample preparation and the Cobas z 480 analyzer for automated amplification and detection following Cobas® *EGFR* Mutation Test kit manual instructions.

#### Ancestry analysis

The ancestry analysis was performed using a set of 46 ancestry-informative markers (AIMs) among the most informative INDELs for Native American (AME), European (EUR), African (AFR), and East Asian (ASN) population groups as previously published^43^. Primer sequences and PCR conditions were previously described^43,44^. Multiplex PCR was performed and the amplified products were submitted to fragment analysis on a 3500xL Genetic Analyzer, ABI capillary electrophoresis system, according to the manufacturer’s instructions. The electropherograms were analyzed and genotypes were automatically assigned using GeneMapper v4.1 (Applied Biosystems).

Ancestry proportions were then assessed using the Structure v2.3.3 software^45,46^ considering the four major population groups as possible contributors to the current genetic background of the Brazilian population. Data from the Human Genome Diversity Panel (HGDP-CEPH) previously demonstrating no significant departures from Hardy-Weinberg equilibrium and linkage equilibrium^43^ were employed as a reference for the ancestral populations and a supervised analysis was performed to estimate ancestry membership proportions of the individuals involved in the present study. Structure v2.3.3 software runs considering K = 4 consisted of 100.000 burnin steps followed by 100.000 Markov Chain Monte Carlo iterations. The option ‘Use population Information to test for migrants’ was used with the Admixture model, considering allele frequencies correlated, and updating allele frequencies using only individuals with POPFLAG = 1.

### Statistical analysis

Clinicopathological factors were used in univariate and multivariate to determine whether the mutations have a significant effect on the parameter. Ancestry proportions were defined as categorical variables according to Lima-Costa *et al*.^47^. The significance of multivariate analysis for association with the presence of the mutations was assessed by the Wald test. Survival analysis was carried out using the Kaplan-Meier method and the Log-rank test. The multivariate analysis was performed by the Cox proportional hazard model to determine whether they have a significant effect on overall survival. All statistical analyses were conducted using SPSS 21.0 (IBM Corp, Armonk, NY, USA) and the level of significance was 5%.

## Supplementary information

Supplementary material

## Data Availability

The data that support the findings of this study are available from Dr. Rui Manuel Reis but restrictions apply to the availability of these data, which were used under ethics committee approval for the current study, and so are not publicly available because of patients’ personal data. Data are however available from the authors upon reasonable request and with permission of the Dr. Rui Manuel Reis (Scientific and Executive Director of the Molecular Oncology Research Center, Barretos Cancer Hospital).
