# Supplementary material for: Mutational profile of Brazilian lung adenocarcinoma unveils association of EGFR mutations with high Asian ancestry and independent prognostic role of KRAS mutations
Source: Sci Rep. 2019 Mar 1;9:3209. doi: 10.1038/s41598-019-39965-x (PMC6397232; doi:10.1038/s41598-019-39965-x)
Supplement: Supplementary file 1 — Supplementary material [file 41598_2019_39965_MOESM1_ESM.pdf]

Title page

Mutational profile of Brazilian lung adenocarcinoma unveils association of *EGFR* mutations with high Asian ancestry and independent prognostic role of *KRAS* mutations

Letícia Ferro Leal[1*], Flávia Escremim de Paula[2*], Pedro De Marchi[3*], Luciano de Souza Viana[3], Gustavo Dix Junqueira Pinto[3], Carolina Dias Carlos[2], Gustavo Noriz Berardinelli[2], José Elias Miziara[4], Carlos Maciel da Silva[4], Eduardo Caetano Albino Silva[5], Rui Pereira[6,7], Marco Antonio de Oliveira[8] , Cristovam Scapulatempo-Neto[5] , Rui Manuel Reis[1,2,9,10]

Affiliations:

[1]Molecular Oncology Research Center, Barretos Cancer Hospital, Barretos, Brazil; [2] Center of Molecular Diagnoses, Barretos Cancer Hospital, Barretos, Brazil; [3]Medical Oncology Department, Barretos Cancer Hospital, Barretos, Brazil; [4]Department of Thoracic surgery, Barretos, Brazil; [5]Department of Pathology, Barretos Cancer Hospital, Barretos, São Paulo, Brazil; [6]Institute of Research and Innovation in Health, University of Porto, Porto, Portugal, [7]Institute of Molecular Pathology and Immunology at the University of Porto (IPATIMUP), Porto, Portugal, [8]Statistics Unity, Barretos Cancer Hospital, Barretos, São Paulo, Brazil; [9]Life and Health Sciences Research Institute (ICVS), Health Sciences School, University of Minho, Braga, Portugal; [10]ICVS/3B's-PT Government Associate Laboratory, Braga/Guimarães, Portugal.

* These authors contributed equally to the present study.

Short title: *EGFR* and *KRAS* mutations in Brazilian lung adenocarcinomas

Corresponding author:

Rui Manuel Reis, PhD

Molecular Oncology Research Center, Barretos Cancer Hospital

Rua Antenor Duarte Villela, 1331; CEP 14784 400, Barretos, S. Paulo, Brazil

Email: ruireis.hcb@gmail.com; Telephone:+551733216600

Supplementary Figure 1

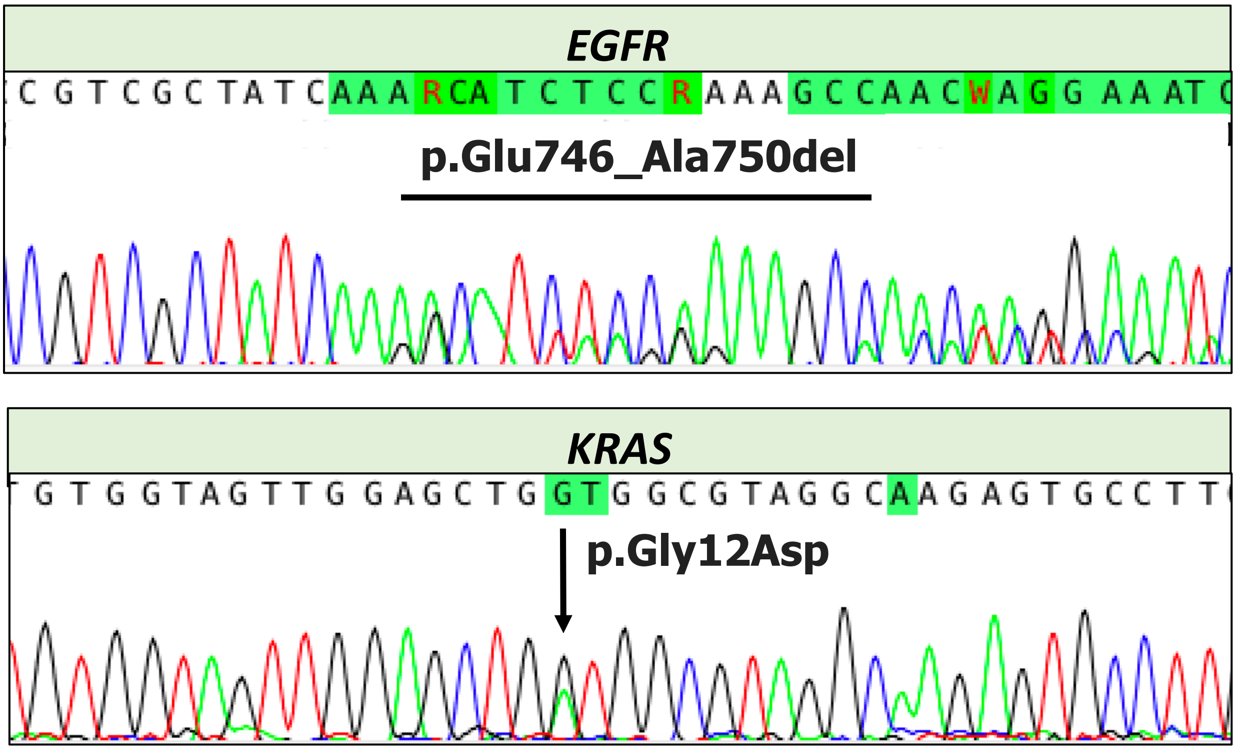

Supplementary Figure 1. Representative electropherogram from the most common *EGFR* (upper panel) and *KRAS* (lower panel) mutations.

Supplementary Figure 2

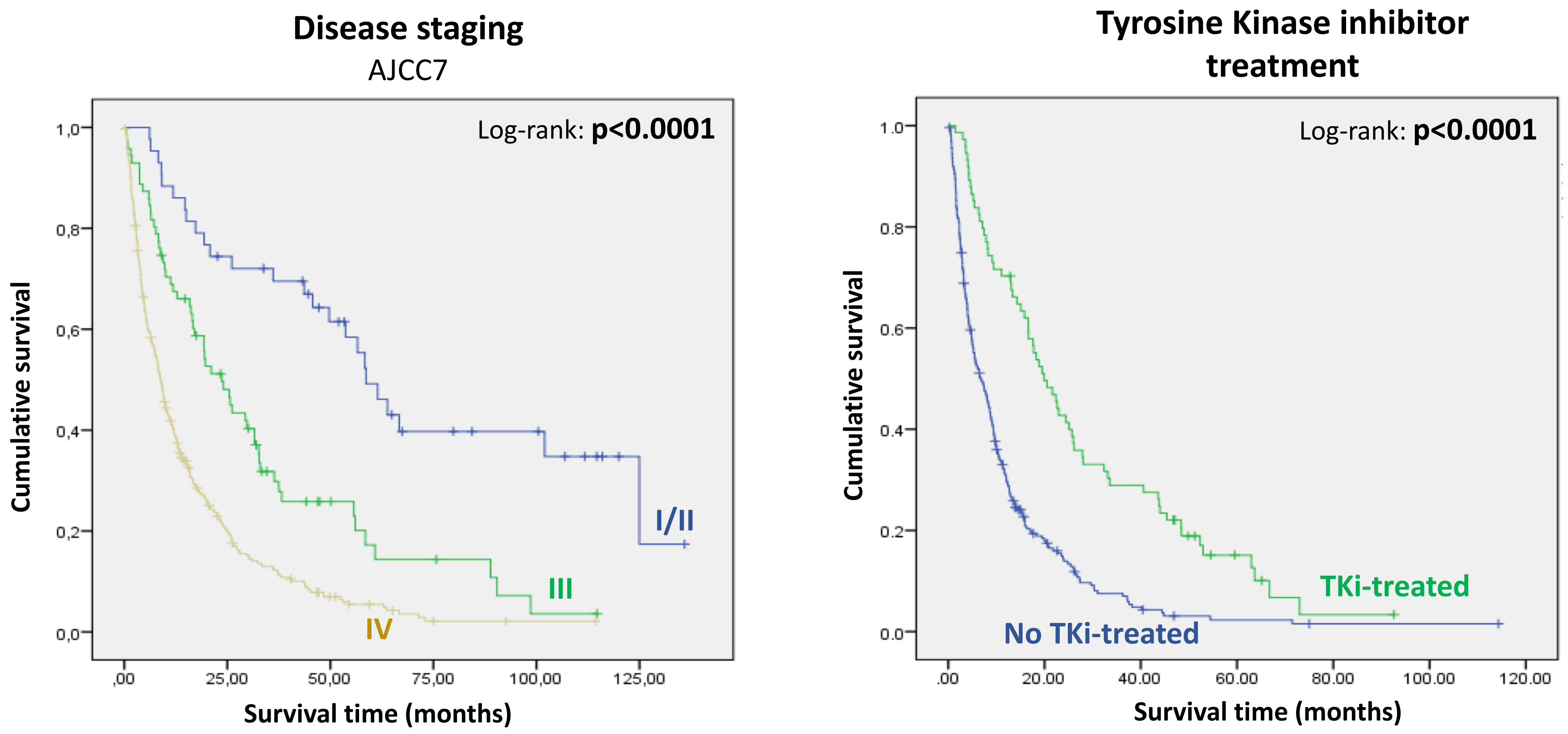

Supplementary Figure 2. Kaplan-Meier curves for overall survival of lung adenocarcinoma patients according to disease staging (left panel; Median OS Stage I/II= 58.6 months; Median OS Stage III = 23.7 months; Stage IV= 8.8 months) and Tyrosine Kinase treatment (right panel; Median OS TKi non-treated= 6.7 months; Median OS TKi-treated= 19.9 months). Survival time is presented in months; p values are related to Log-rank test results.

Supplementary Table 1. Frequency of all EGFR mutations detected in the present study according to exon distribution.

Exon	**n**	**(%)**	**n (%) Total**
**18**			
p.Gly719Ser	1	50	2 (1.9%)
p.Gly719Ala	1	50	
**19**			
p.Glu746_Ala750del	31	58.5	53 (49.1%)
p.Glu746_Ser752delinsVal	4	7.5	
p.Glu746_Thr751delinsAla	1	1.9	
p.Leu747_Ala750delinsPro	2	3.8	
p.Leu747_Pro753delinsGln	1	1.9	
p.Leu747__Pro753delinsSer	7	13.2	
p.Leu747_Ser752del	2	3.8	
p.Leu747_Thr751del	3	5.7	
p.Ser752_Ile759del	1	1.9	
p.Asp761Tyr	1	1.9	
**20**			
p.Gln761_Ala763dup	2	15.4	13 (12.0%)
p.Ser768_Asp770dup	1	7.7	
p.Ser768Ile	1	7.7	
p.Asp770_Asn771insTyr	1	7.7	
p.Asp770_Val774dup	1	7.7	
p.Ans771_His773dupAsnProHis	1	7.7	
p.Pro772_His773dup	1	7.7	
p.His773_Val774dup	1	7.7	
p.Gly810Asp	1	7.7	
p.Thr790Met*	3	23.1	
**21**			
p.Glu829Gln	1	2.5	40 (37.0%)
p.Leu833Val	1	2.5	
p.Val834Leu	1	2.5	
p.His850Asp	1	2.5	
p.Leu858Arg*	32	80	
p.Ala859Thr	1	2.5	
p.Leu861Gln*	3	7.5	

**These mutations may appear simultaneously with other EGFR mutations.*

Supplementary Table 2. Frequency of all *KRAS* mutations detected in the present study according to codon distribution.

Codon	n	(%)	n (%) Total
**12**			
p.Gly12Cys	32	40%	80 (88.9%)
p.Gly12Val	21	26.25%	
p.Gly12Asp	19	23.75%	
p.Gly12Ala	5	6.25%	
p.Gly12Ser	2	2.5%	
p.Gly12Phe	1	1.25%	
**13**			
p.Gly13Cys	6	60%	10 (11.1%)
p.Gly13Asp	1	10%	
p.Gly13Glu	1	10%	
p.Gly13Ser	1	10%	
p.Gly13Val	1	10%	

Supplementary Table 3. Ancestry background categorization according to tercile based on the percentage proportions for all four ethnic groups.

Ancestry component	Low	Intermediate	High
**ASN**	< 0.028	0.028 - 0.055	> 0.055
**AFR**	< 0.027	0.027 - 0.125	> 0.125
**EUR**	< 0.698	0.698 - 0.865	> 0.865
**AME**	< 0.029	0.029 - 0.058	> 0.058

Cut off values were determined according to tercile categorization.

Supplementary Table 4. Univariate analysis of the association between clinicopathological characteristics, ancestry background and clinical outcome of lung adenocarcinoma patients.

Variables	Parameters	*EGFR*				*KRAS*			
		n	WT (%)	Mutated (%)	p-value	n	WT (%)	Mutated (%)	p-value
Age	≤ 61 years	239	78.7	21.3	0.50	238	78.2	21.8	0.41
	> 61 years	205	75.6	24.4		204	81.4	18.6	
Gender	Male	232	84.5	15.5	**<0.0001**	230	78.7	21.3	0.64
	Female	212	69.3	30.7		212	80.7	19.3	
Self-reported race*	White	342	76.9	23.1	0.40	340	79.7	20.3	0.46
	Brown	64	81.2	18.8		64	81.2	18.8	
	Black	20	85.0	15.0		20	85.0	15.0	
	Yellow	7	57.1	42.9		7	57.1	42.9	
Smoking	Never smoker	135	51.1	48.9	**<0.0001**	135	91.1	8.9	**<0.0001**
	Current Smoker	171	90.6	9.4		169	75.7	24.3	
	Former smoker	131	85.5	14.5		131	73.3	26.7	
Alcohol consuming	Never	272	70.6	29.4	**0.002**	271	83.0	17.0	0.14
	Current	114	86.0	14.0		113	74.3	25.7	
	Former	35	85.7	14.3		35	82.9	17.1	
Disease staging	I	28	75.0	25.0	0.17	28	75.0	25.0	0.11
	II	15	80.0	20.0		15	60.0	40.0	
	III	71	87.3	12.7		71	74.6	25.4	
	IV	329	75.1	24.9		327	82.0	18.0	
Metastasis at diagnosis	No	114	83.3	16.7	0.07	114	72.8	27.2	0.09
	One site	129	79.1	20.9		127	80.3	19.7	
	Multiple sites	200	72.5	27.5		200	83.0	17.0	
PS ECOG	0	46	63.0	37.0	**0.03**	46	67.4	32.6	0.173
	1	233	75.5	24.5		231	81.4	18.6	
	2	73	80.8	19.2		73	82.2	17.8	
	3 or 4	88	85.2	14.8		88	79.5	20.5	
Loss of weight**	No	209	75.6	24.4	0.78	208	77.4	22.6	0.37
	< 10 %	145	75.9	24.1		145	83.4	16.6	
	> 10%	69	79.7	20.3		68	80.9	19.1	
Differentiation grade	G1	17	82.4	17.6	0.90	17	88.2	11.8	0.68
	G2	56	80.4	19.6		56	76.8	23.2	
	G3	82	75.6	24.4		82	84.1	15.9	
	G4	7	85.7	14.3		7	85.7	14.3	
ASN ancestry	Low	143	81.8	18.2	**0.03**	143	74.1	25.9	0.11
	Intermediate	139	81.3	18.7		139	79.9	20.1	
	High	145	70.3	29.7		145	84.1	15.9	
AFR ancestry	Low	142	77.5	22.5	0.97	142	78.9	21.1	0.73
	Intermediate	141	77.3	22.7		141	81.6	18.4	
	High	144	78.5	21.5		144	77.8	22.2	
EUR ancestry	Low	141	73.0	27.0	0.14	141	79.4	20.6	1.00
	Intermediate	141	77.3	22.7		141	79.4	20.6	
	High	145	82.8	17.2		145	79.3	20.7	
AME ancestry	Low	151	76.8	23.2	0.41	151	82.8	17.2	0.45
	Intermediate	131	81.7	18.3		131	77.1	22.9	
	High	145	75.2	24.8		145	77.9	22.1	

*n, number of patients; WT, wild-type; p-value: significance of t test.; ASN, Asian ancestry; AFR, African ancestry; EUR, European ancestry; AME, Amerindian ancestry; PS ECOG, performance status ECOG (Eastern Cooperative Oncology Group); Ref., reference group. *Self-reported race according to Brazilian Institute of Geography and Statistics (IBGE). **Loss of weight <10% and >10% of total body weight. Significant associations are indicated in bold.*

Supplementary Table 5. TKi response regarding *EGFR* mutations per exon.

TKi response vs. EGFR mutation (per exon)						
			TKi response			Total
			**CR/PR**	**SD**	**DP**	
***EGFR* mutations per exon**	**Exon 19**	*n*	29	5	2	36
		n (%)	85.3%	55.6%	22.2%	69.2%
	**Exon 20**	n	1	0	5	6
		n (%)	2.9%	0.0%	55.6%	11.5%
	**Exon 21**	n	4	4	2	10
		n (%)	11.8%	44.4%	22.2%	19.2%
Total		n	34	9	9	52

TKi, tyrosine kinase inhibitor; CR, complete response; PR, partial response; SD, stable disease; DP, disease progression; n, number of cases in each category. **Fisher's exact test: $p<0.0001$**

Supplementary Table 6. Detailed information about surgical resection.

	n	Surgery type	Additional information
Surgical resection	51	Lobectomy n=46	IA (n=8); IB (n=12)<br>IIA (n=7); IIB (n=4)<br>IIIA (n=12); IIIB (n=1)<br>IV (n=7)
		Segmentectomy n=4	
		Pneumectomy n= 1	

Supplementary Table 7. Summary of treatment regimens in *EGFR*-mutated and *EGFR* wild-type patients.

	**n**	**Adjuvant** (n)	**Neoadjuvant/ induction** (n)	**TKi** (n)	**≥ 1 palliative line** (n)	**Number of palliative lines** (average)
EGFR-mutated	101	1	4	71	87	2.29
EGFR wild-type	343	9	31	24	188	1.83

n, number of patients; TKi, patients tyrosine kinase inhibitor

Supplementary Table 8. Detailed information about cytotoxic treatment regimens in *EGFR*-mutated patients.

	**Cytotoxic chemotherapy regimens**
Adjuvant therapy	Cisplatin + vinorelbine
Neoadjuvant/induction therapy	Carboplatin + paclitaxel
1st cytotoxic palliative line	Carboplatin + paclitaxel
	Carboplatin + gemcitabine
	Cisplatin + vinorelbine
	Cisplatin + pemetrexed
	Gemcitabine
	Paclitaxel

Supplementary Table 9. Detailed information about cytotoxic treatment regimens in *EGFR* wild-type patients.

	**Cytotoxic chemotherapy regimens**
Adjuvant therapy	Cisplatin + vinorelbine
	Carboplatin + Paclitaxel
Neoadjuvant/induction therapy	Carboplatin + Paclitaxel
	Carboplatin + Etoposide
	Cisplatin+Etoposide
1st cytotoxic palliative line	Carboplatin+Paclitaxel
	Carboplatin+Docetaxel
	Carboplatin+Gencitabine
	Cisplatin+ Etoposide
	Cisplatin+Gencitabine
	Cisplatin+Placlitaxel
	Cisplatin + Pemetrexed
	Vinorelbine
	Gencitabine
	Paclitaxel
